# Chasing the Wrong Door for 30 Years: Is Suzetrigine the Key to Selective Pain Relief?

**DOI:** 10.3390/medicina62091698

**Published:** 2026-09-04

**Authors:** Robert Ancuceanu, Athena Ribigan, Adriana-Iuliana Anghel, Mihaela Dinu

**Affiliations:** 1 Faculty of Pharmacy, Department of Pharmaceutical Botany and Cell Biology, Carol Davila University of Medicine and Pharmacy, 050474 Bucharest, Romania; robert.ancuceanu@umfcd.ro (R.A.); adriana.anghel@umfcd.ro (A.-I.A.); mihaela.dinu@umfcd.ro (M.D.); 2 Faculty of Medicine, Department of Clinical Neurosciences, Carol Davila University of Medicine and Pharmacy, 020021 Bucharest, Romania; 3 Neurology Department, University Emergency Hospital Bucharest, 050098 Bucharest, Romania

**Keywords:** voltage-gated sodium channels, NaV1.7, NaV1.8, suzetrigine, pain therapy

## Abstract

For a long time, pain management has relied on relatively old opioids and broad-spectrum non-opioids, which display efficacy gaps and non-negligible safety risks. This review explores why voltage-gated sodium channels, particularly NaV1.7 and NaV1.8, emerged as prime targets for pain relief, and why early development efforts failed. NaV1.7 was initially considered a promising target due to its role in nociception and genetic evidence linking it to pain disorders. However, clinical trials of selective NaV1.7 inhibitors have repeatedly failed. We explore the reasons for these failures and discuss the clinical shortcomings of non-selective blockers and early NaV1.7 inhibitors. In contrast, NaV1.8 has emerged as a much stronger drug target, because it is mostly limited to peripheral nerves, directly drives continuous pain signaling, and shows less apparent functional redundancy in specific nociceptive axonal compartments. Nevertheless, compensatory mechanisms involving NaV1.7, NaV1.9, and other conductances remain context-, tissue-, and disease-dependent. Suzetrigine, a highly selective inhibitor of this channel, recently gained regulatory approval for acute pain, marking a major breakthrough in non-opioid options, even though its Phase 3 efficacy was comparable to hydrocodone/acetaminophen rather than superior to it. Its clinical success is built on high selectivity, excellent pharmacokinetics, and a smart trial design that focused specifically on standardized postoperative pain models. While suzetrigine shows that NaV1.8 can be successfully targeted, it represents a regulatory and mechanistic victory rather than a complete replacement for opioids in daily practice. Progress in this field will ultimately require precise targeting, better pharmacology, and trials that better match patient phenotypes.

## 1. Introduction: The Longstanding Crisis in Analgesic Drug Development

Over the last half-century, the clinical landscape of pain management has barely evolved. Comparing a contemporary pharmacology textbook to one from fifty years ago highlights a sobering reality: despite decades of research, the emergence of truly novel, mechanistically distinct analgesics has been virtually non-existent. This prolonged innovation drought underscores a structural crisis in drug development, leaving clinicians entirely reliant on opioids to combat severe acute, surgical, and malignant pain. In 2020, opioids still appeared “to be most promising among current approaches in the development of analgesics” [1]. While undeniably effective, opioids carry a heavy clinical toll; their widespread deployment is fundamentally limited by important side effects like respiratory depression [2], sedation, constipation [3], tolerance, and addiction, culminating in a global addiction epidemic [4]. Resolving this crisis is a big challenge, as it stems from a complex intersection of heterogenous factors and vulnerabilities. These go from genetic variations in opioid receptor sensitivity [5] to the socio-economic conditions that shape prescribing practices and substance abuse [6].

Whereas opioids remain key medicines in managing acute and cancer pain, their role in chronic nonmalignant pain management has become controversial due to concerns over their potential misuse and dependence, and uncertainties regarding long-term efficacy [7,8]. Achieving effective pain relief while limiting the harmful risks associated with opioid use remains a daunting therapeutic challenge. This underscores the pressing need for novel analgesic agents capable of providing good pain control without causing significant adverse effects [9]. To address these challenges, various approaches are explored, such as improvements in the pharmacokinetic and pharmacodynamic properties of available agents [10], as well as the development of abuse-deterrent formulations [11]. There is also an increased interest toward personalized medicine strategies that leverage molecular and genetic profiling to optimize individual patient care [12].

Non-opioid analgesics, in their turn, are often effective only for mild to moderate pain, whereas their efficacy is substantially lower in severe pain conditions and in chronic pain. They often fail to provide adequate relief in neuropathic pain (many chronic pain states are neuropathic) [13], cancer pain [14], and centralized pain syndromes such as fibromyalgia [15]. Paracetamol shows relatively weak efficacy across many pain conditions, sometimes offering benefits close to placebo [16]. NSAIDs exert their effects through inhibition of cyclooxygenase (COX-1 and COX-2), but this mechanism produces substantial off-target toxicity: COX-1 blockade diminishes protective gastric prostaglandins, leading to ulceration, gastrointestinal bleeding, and perforation [17]. NSAIDs also reduce renal prostaglandin synthesis, potentially causing acute kidney injury [18], sodium retention, worsening hypertension [18], and at high doses, chronic kidney damage [19]. Selective COX-2 inhibitors and some nonselective NSAIDs additionally carry significant cardiovascular risks, including myocardial infarction, stroke, heart failure, and thrombotic events [20]. In addition, pain is biologically complex and involves inflammatory mediators and activation of peripheral nociceptors [21], immune-cell signaling (the old distinction between ‘neuronal’ and ‘immune’ ion channels and receptors is increasingly becoming blurred) [22], ion channel dysfunction [23], and left unchecked, spinal sensitization [24] and central sensitization [25]; such complexity is not adequately addressed by merely inhibiting COX, as usually done by conventional non-opioids.

To overcome these therapeutic limitations of both opioid and non-opioid analgesics, research efforts have shifted toward uncovering new non-opioid analgesic targets and developing novel pharmacological therapies. These are tailored to distinct pain etiologies, including inflammatory, neuropathic, and nociplastic pain. Such novel targets span the entire nociceptive pathway [26,27], beginning with tissue-level inflammatory and biochemical modulators including proteases and matrix metalloproteinases [28,29], protease-activated receptor 2 (PAR-2) [30], various chemokines and chemokine receptors [31], and pro-resolution lipid mediators (such as resolvin D1 and protectin D1) [32]. At the nociceptor membrane, key molecular transducers and propagators targeted include TRP ion channels (particularly TRPV1) [33], voltage-gated sodium channels (Na_v_ channels) [34], and voltage-gated calcium channels (specifically N-type and T-type) [35]. Finally, upstream synaptic transmission and central signaling targets encompass glutamate receptors (particularly NMDA) [36] and WNT inhibitors [37]. Because nociception relies heavily on neuronal excitability, voltage-gated sodium channels (VGSCs) have emerged as critical molecular targets in pain research.

Over time, a mounting body of evidence has emerged suggesting that therapeutic success in pain management, regardless of the treatment used, depends heavily on the phenotype of the pain being treated [1,2,3,4,5,6]. Therefore, distinguishing between these types of pain is essential:

(a) **Nociceptive pain** (e.g., acute postoperative pain). It is caused by harmful mechanical, thermal, or chemical stimulation of intact peripheral nociceptors with a high threshold, following tissue trauma. It can be somatic or visceral and presents either as a sharp, fast pain transmitted via A-fibers, or as a slower, dull pain transmitted via C-fibers [38,39].
(b) **Inflammatory pain** (often regarded as a subtype of nociceptive pain [40]). It involves tissue damage and inflammatory mediators (e.g., prostaglandins, cytokines, chemokines, bradykinin, protein kinase A) that cause nociceptive sensitization and a decrease in activation thresholds [41,42,43].
(c) **Peripheral neuropathic pain** (e.g., diabetic neuropathy, chemotherapy-induced neuropathy, or postherpetic neuropathy). It results from direct injury or a disease affecting the peripheral somatosensory nerves, leading to ectopic discharges in the injured nerves and hyperexcitability of the peripheral fibers. Generally, the smaller fibers are affected (the myelinated A-beta and delta fibers and the unmyelinated C fibers) [44,45].
(d) **Central neuropathic pain.** It occurs as a result of lesions or dysfunctions of the central nervous system (e.g., spinal cord injury, post-stroke pain, multiple sclerosis), due to increased reactivity of nociceptive neurons (a process known as central sensitization). This is a maladaptive process in which the brain and spinal cord misinterpret the signals they receive and spontaneously generate painful sensations [45,46].
(e) **Nociplastic pain.** Conceptualized most recently (2016) [47], it is characterized by altered nociceptive processing and central sensitization without clear evidence of tissue or somatosensory damage [47]. It is multifocal in nature, results from increased sensory processing in the central nervous system and alterations in pain modulation. It manifest itself as widespread or disproportionate pain, with associated central symptoms (fatigue, sleep disturbances, cognitive difficulties, and mood problems) [47,48].

Extrapolating therapeutic efficacy across these different phenotypes is problematic because the relative contribution of therapeutic targets and the response to treatment may vary depending on the predominant mechanism of pain (nociceptive, peripheral or central neuropathic, or nociplastically mediated) [49]. In addition, these phenotypes are not mutually exclusive; nociceptive, neuropathic, and nociplastically mediated components may coexist in the same patient, limiting the extrapolation of results across phenotypes [50].

## 2. Why Sodium Channels Became a Major Analgesic Target

Throughout this review, mechanistic findings are drawn from diverse experimental systems. These include rodent models, human DRG neurons, and clinical trials, and these sources differ in translational weight; where relevant, the specific evidence type is indicated to clarify the strength and applicability of each conclusion.

VGSCs play a fundamental role in electrical signaling within excitable tissues and have been implicated in a broad and increasingly large array of pathological conditions [51]. They are spread across both prokaryotic domains and all four eukaryotic kingdoms. Across most of these organisms, their main physiological role is to initiate propagated action potentials upon minor depolarization of the membrane [52]. At the usual resting membrane potential of peripheral nociceptors, about −60 mV, these channels remain closed.

**Figure 1:**
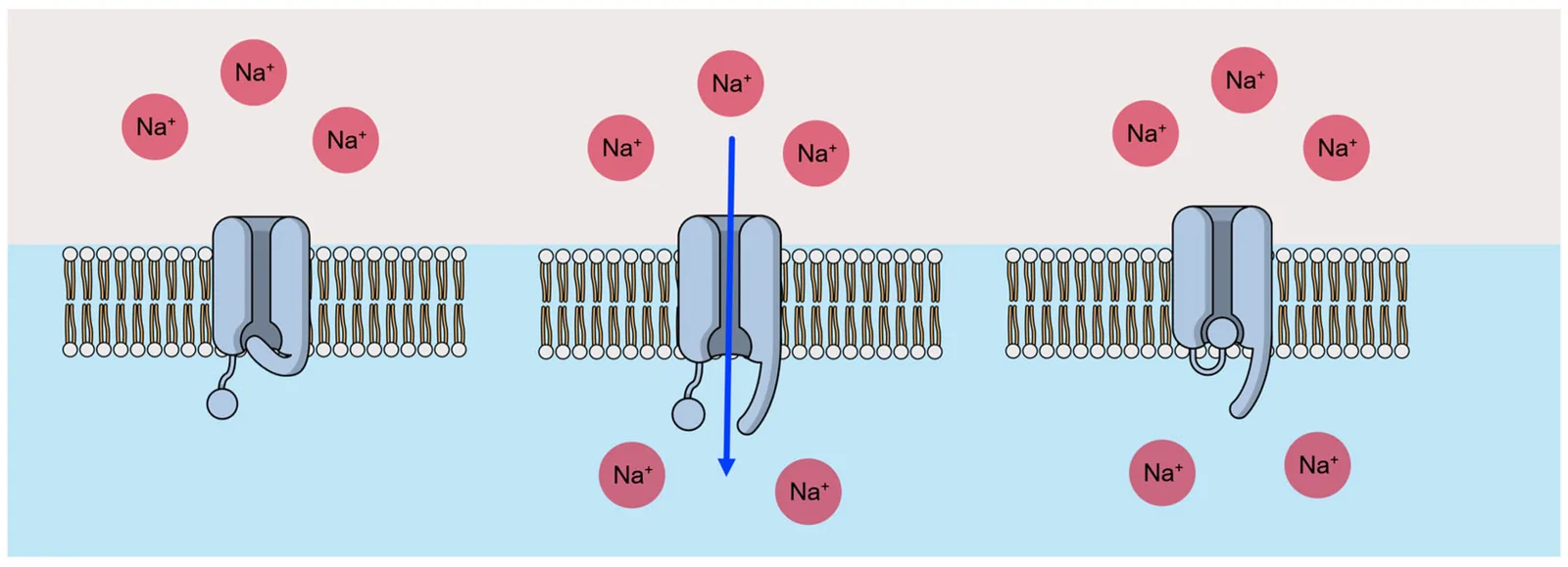
Graphical representation of the three states of sodium channels: resting state (**left**); activated/open state (**middle**); inactivated state (**right**). Before the inactivation gate can reset and reopen for future action potentials, the channel needs to revert to its hyperpolarized resting state (Figure created with Mind the Graph—mindthegraph.com).

Nav channels cycle through three main functional states: closed (resting), open (conducting), and inactivated (nonconducting). A wide array of natural and synthetic Nav ligands exhibit varying binding affinities depending on the specific conformation of the channel, and they are said to be state dependent. Conversely, state independent modulators interact with the channel uniformly, showing no significant preference for any particular conformational state [53]. Truly state-independent inhibition appears to be uncommon among clinically relevant small-molecule sodium channel blockers [54]. When the membrane depolarizes, VGSCs open (*activation*), allowing a swift influx of sodium ions that further drives the membrane depolarization toward the sodium equilibrium potential (~+60 mV in neurons). These channels close within milliseconds, but their inactivation is often incomplete, leaving a small, persistent sodium current that gradually decays over tens of seconds [51]. Channel closure that occurs during sustained membrane depolarization is referred to as *inactivation*. Both activation and inactivation, as well as the coupling between these processes depend on membrane voltage, whereas the subsequent conformational rearrangements proceed independently of voltage changes [55] (Figure 1).

The voltage-gated sodium channel (VGSC) proteins are encoded by a gene family consisting of nine homologous members (SCN1A to SCN5A and SCN8A to SCN11A). These genes encode the alpha subunits of Na_V_1.1 to Na_V_1.9 VGSCs. Each α-subunit typically co-assemble with one or two of the β-subunits, in their turn encoded by SCN1B to SCN4B. SCN6A and SCN7A, however, encode a structurally related Nax channel, which is an outlier, because it is not activated by membrane depolarization. Instead, Nax is activated by fluctuations in extracellular sodium concentrations while operating in close physical association with the Na-K ATPase pump [51,56].

Voltage-gated sodium channel α subunits are distributed in a tissue-dependent manner. The Nav1.1, Nav1.2, and Nav1.3 subtypes occur primarily in the central nervous system, while Nav1.6 is found in both central and peripheral nervous tissue. In contrast, NaV1.7, NaV1.8, and Nav1.9 are largely confined to the peripheral nervous system. Separately, Nav1.4 is highly expressed in skeletal muscle, and Nav1.5 predominates in cardiac muscle [53].

All currently identified Nav channel isoforms are categorized based on their susceptibility to tetrodotoxin (TTX), a guanidinium toxin derived from pufferfish. Subtypes Nav1.1, Nav1.2, Nav1.3, Nav1.4, Nav1.6, and NaV1.7 are effectively obstructed by TTX at low nanomolar levels, thus designating them as TTX-sensitive (TTX-S). Conversely, Nav1.5, NaV1.8, and Nav1.9 require substantially higher, micromolar concentrations for inhibition, which classifies them as TTX-resistant (TTX-R) [53,57,58].

Pain signals originate in primary sensory neurons, which have peripheral endings in the skin or internal organs and central endings in the spinal cord or the brainstem. When such neurons detect a harmful stimulus, they trigger an action potential that travels to the neuron′s central terminal, prompting neurotransmitter release, activating the next neuron in the pathway, and ultimately relaying pain information to the brain. Nav1.3, NaV1.7, NaV1.8, and Nav1.9 play a central role in this nociceptive signaling process [53]. NaV1.7, NaV1.8, and Nav1.9 are mainly found in peripheral rather than central neurons, and each has been connected to hereditary pain conditions in humans [57,59].

Loss-of-function recessive mutations in NaV1.7 (also known as PN1) were found to cause Congenital insensitivity to pain (CIP) [60]. As anticipated by the authors of that discovery, it stimulated intensive research on this sodium channel isoform as a promising target for developing novel analgesics that, in principle, could provide pain relief without significant adverse effects. This was also supported by studies on the naked-mole rats showing that this species is insensitive to acid-related pain, and that the physiological basis for this insensitivity consists in a NaV1.7 mutation [61,62]. However, not all pain forms are dependent exclusively on the expression of this Nav channel. For instance, patients with loss-of-function mutations in the gene encoding it, while pain-insensitive, still can suffer from neuropathic pain [63] and similar is available for chemotherapy-induced neuropathic pain in mice [64].

NaV1.8 is predominantly expressed in neurons of the dorsal root ganglion (DRG) and trigeminal ganglion, which are key mediators of pain signal transmission [65]. It is absent from the CNS (including spinal cord or brain) and it was not detected outside neuronal tissues, being absent, for instance from heart or skeletal muscles [66]. Non-clinical experimental evidence based on loss-of-function NaV1.8 mutated mice and other non-clinical data, as well as human data linking NaV1.8 mutations to small-fiber neuropathy support a role of this channel as a contributor to pain [66]. Among VGSCs, NaV1.8 has been strongly associated with both the onset and maintenance of chronic pain across numerous pathological conditions, including visceral disorders [65].

NaV1.9 is a key modulator of DRG neuron excitability, having an influence on how pain develops and persists. It helps set the resting membrane potential of DRG neurons by generating a large persistent sodium current [67]. Genetic variants in NaV1.9 (encoded by *SCN11A*) have been linked to several inherited pain disorders, including episodic pain syndromes and painful neuropathies [68,69,70].

The NaV1.7, NaV1.8, and Nav1.9 are primarily expressed in small nociceptive neurons within the dorsal root ganglia (DRGs) [71] and can be viewed as a functional unit. They operate in a coordinated, partially overlapping manner within small-diameter DRG to generate the complex process of peripheral nociception. In the classical model, each channel has a distinct role: Nav1.9 regulates baseline excitability of DRG neurons by producing a large persistent current [70]; NaV1.7 functions as a “threshold” channel that amplifies small depolarizations, whereas NaV1.8 carries the majority of the inward current during action potentials in many nociceptive DRG neurons [72,73,74]. Following nerve injury or inflammation, DRG neurons frequently display upregulated or functionally modified NaV1.7 and NaV1.8 expression and kinetics. This lowers the firing threshold and promotes spontaneous or ectopic discharges, which are central features of neuropathic pain [75].

**Figure 2:**
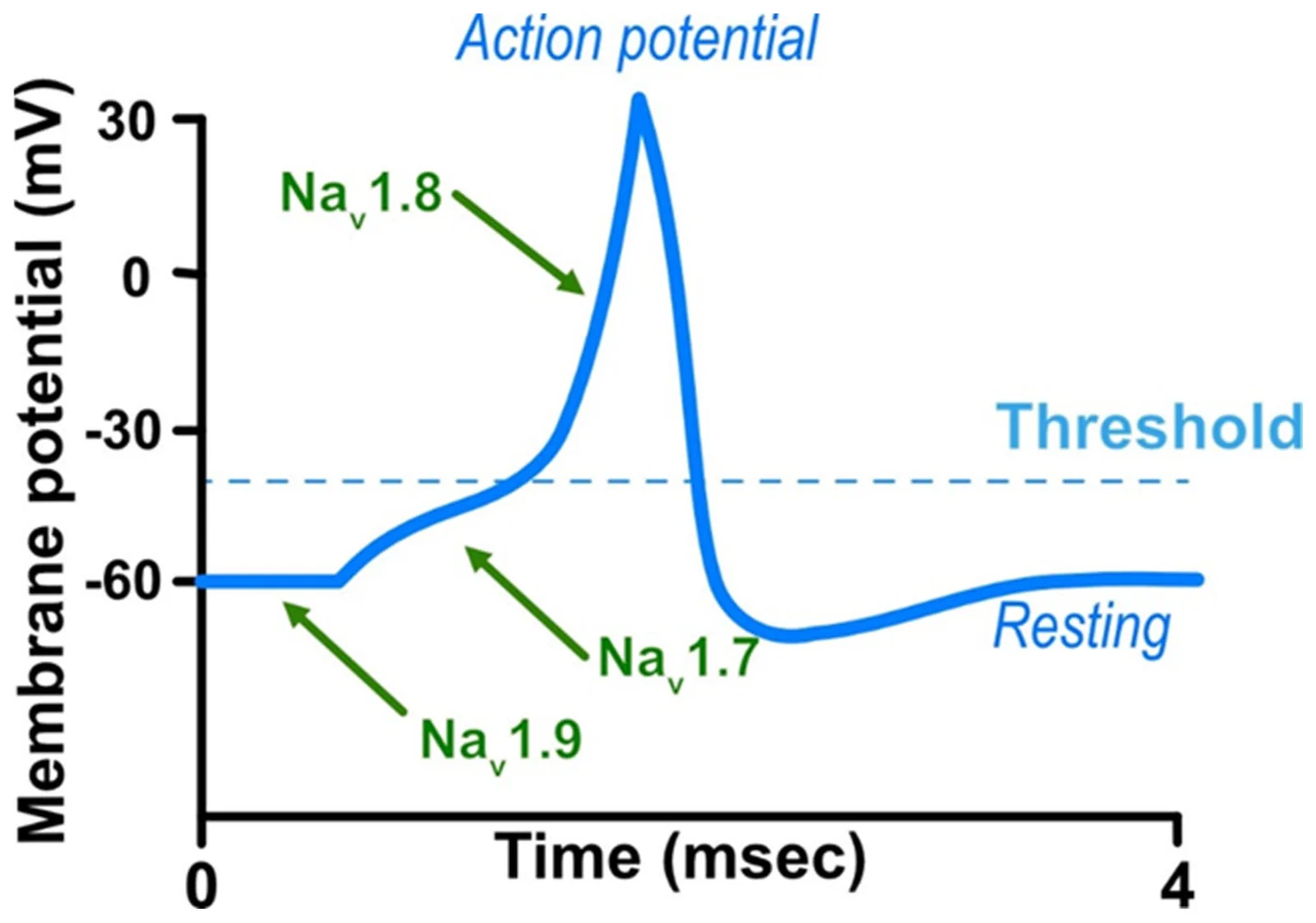
Role of NaV1.7, 1.8 and 1.9 in action potential generation. Schematic showing how different voltage-gated sodium channel subtypes coordinate to depolarize the membrane potential past the threshold level to generate a neuronal spike.

This functional separation of roles has been challenged by recent gating and modeling data, which demonstrate a much more integrated cooperation between the channels. Specifically, NaV1.9 contributes not only to subthreshold excitability but also supports the action potential upstroke and shoulder. Computational modeling further indicates that NaV1.7 is the principal determinant of action potential threshold, whereas NaV1.8 remains the major contributor to the action potential upstroke and sustained repetitive firing [76]. Thus, while the three channels do correspond broadly to distinct phases of the action potential, they function as an interdependent team, rather than three distinct, non-overlapping agents (Figure 2).

The exclusive peripheral expression of NaV1.7, NaV1.8, and NaV1.9 makes them ideal targets for pain relief. By selectively blocking these isoforms, new therapies could theoretically interrupt pain signals at their origin in the periphery, sidestepping the central nervous system toxicities (e.g., sedation) and cardiac risks associated with broader sodium channel inhibitors.

## 3. Why Non-Selective Blockade Failed

Non-selective sodium channel inhibitors, largely repurposed anticonvulsants and antiarrhythmics such as carbamazepine, oxcarbazepine, eslicarbazepine, phenytoin, lamotrigine, lacosamide, valproate, topiramate, lidocaine, mexiletine, flecainide and (particularly in Chinese medicine) bulleyaconitine A, were the earliest attempts to modulate Nav channels for analgesia (Table 1). Although these agents are effective in epilepsy and certain neuralgias, they have often failed in generalized neuropathic pain trials. Their failure is rooted in the interplay of three factors: mechanistic mismatches, lack of isoform specificity, and dose-limiting systemic toxicity.

### 3.1. Mechanistic Mismatch with Neuropathic Pain Physiology

Repurposed older therapies inhibiting sodium channels often act as *state-dependent* sodium-channel blockers, binding with high affinity to the inactivated state of the channel. This makes them highly appropriate for epilepsy (where most of them are used), because they selectively suppress high-frequency, synchronized pathological firing while leaving normal neuronal activity largely unaffected [97]. Nevertheless, neuropathic pain is not always associated with high-frequency burst activity. Numerous peripheral pain conditions arise instead from slow, sustained currents or alterations in resting membrane potential, processes predominantly governed by Nav1.9 [98] and low-frequency ectopic pacemaking within injured axons [99].

### 3.2. Lack of Nav Isoform Selectivity

Carbamazepine, oxcarbazepine, and eslicarbazepine exhibit no meaningful selectivity among Nav isoforms [78]. Their high affinity for the inactivated state applies broadly across Nav1.1–Nav1.9, including isoforms essential for normal CNS, cardiac, and skeletal muscle function. Most other legacy blockers have also almost no selectivity. Consequently, attempts to achieve analgesic concentrations inevitably lead to off-target effects. From the anatomical territory distribution of different isoforms, it is hypothesized that inhibition of Nav1.4 and Nav1.5 produces musculoskeletal and cardiovascular adverse effects. Because Nav1.6 is highly expressed in myelinated sensory and motor neurons, its blockade is expected to result in paresthesia and muscle weakness. Likewise, inhibition of centrally expressed sodium channel isoforms, including Nav1.1, Nav1.2, and Nav1.3, is thought to carry the potential for cognitive disturbances and seizure activity [100,101]. Most of these AEs are known for the non-selective Nav inhibitors, although many of them have also additional off-target effects, e.g., calcium channel inhibition and GABA transaminase inhibition [102] (valproate) or glutamine release inhibition (lamotrigine) [103]. The anatomical distribution makes peripheral analgesia unattainable without provoking central or cardiac toxicity.

### 3.3. Systemic Toxicity Prevents Dose Escalation

Most of these medications are characterized by a narrow therapeutic window and a high incidence of severe adverse effects. The latter are related to their effects on Nav subtypes in the central nervous system and heart, including dizziness, sedation, seizures, and cardiotoxicity (Table 1). Most often current guidelines do not recommend them for neuropathic pain treatment, likely due to their unfavorable side effect profiles and limited effectiveness evidence. The exception is tricyclic antidepressants (TCAs), which are not pure sodium channel blockers but instead act on multiple receptors and channels, such as serotonergic, noradrenergic, dopaminergic, histaminergic, muscarinic, and calcium channels, as well as inhibiting neurotransmitter reuptake [100]. Escalating doses to the levels required to saturate peripheral pain fibers in clinical practice is likely to trigger dangerous central and cardiac toxicity before reaching the analgesic effects.

### 3.4. Systemic Toxicity Drives Patient Dropout Rates

Another factor that probably contributed substantially to the failure of non-selective Nav channel blockers is the fact that chronic pain necessitates extended treatment, and any therapeutic agent must demonstrate acceptable safety and tolerability over prolonged periods. Non-selective sodium channel blockers have often failed in large-scale trials, primarily because systemic toxicity drives patient dropout rates to unacceptably high levels, as discussed above for mexiletine (where about half of all patients stop therapy before two months of treatment [95]) or carbamazepine (where 66% reported at least one adverse reaction, compared to 27% in the placebo group [80]).

To conclude, the ineffectiveness of non-selective sodium channel blockers can be attributed to a combination of biophysical, anatomical, and pharmacokinetic limitations. Most of these drugs were primarily designed to treat epilepsy, not peripheral neuropathic or chronic pain. Their inability to target specific isoforms leads to unintended toxic effects, and their widespread distribution in the body hinders the attainment of therapeutic levels at nociceptors. While some may still be useful for specific conditions like trigeminal neuralgia, the overall failure of this class of drugs highlights the necessity for Nav inhibitors that are isoform-specific and restricted to peripheral areas. The disappointing results of non-selective blockers sparked excitement and high hopes for NaV1.7-selective agents. Yet, despite robust genetic support, these new class inhibitors have not achieved the expected clinical success.

## 4. The NaV1.7 Selective Inhibitors: Repeated Clinical Failure Despite High Initial Expectations

NaV1.7 is abundant in DRG nociceptors and other peripheral neurons. Its activation by tiny, slow depolarizations allows it to boost weak stimuli, giving it a key role in setting neuronal excitability [104]. An important body of non-clinical evidence established their importance in pain genesis. Genetic ablation of these channels, achieved either systemically or in a tissue-restricted manner, was shown experimentally to elevate pain resistance for a variety of pain forms (mechanical, inflammatory, heat-induced) [105]. In rats with STZ-induced diabetes, the dorsal root ganglia contain higher amounts of the sodium channel proteins NaV1.7 and NaV1.3, whereas normalization of NaV1.7 expression significantly reduced pain-related behaviors (an effect not observed for formalin-induced pain) [106]. In NaV1.7 knockout mice, mechanical sensitivity and general movement remained unaffected, while the animals exhibited a total absence of pain responses to tactile, thermal, and chemical stimuli (and were also unable to detect odors). Furthermore, these mice did not display pain behaviors after peripheral administration of nonselective sodium channel activators, failed to develop thermal hyperalgesia from complete Freund’s adjuvant, and were insensitive to intradermal histamine injection [107].

Gain-of-function missense mutations affecting a single allele of the *SCN9A* gene have been identified as the root cause of several rare, severe pain disorders, mainly inherited erythromelalgia (IEM) [104,108,109] and paroxysmal extreme pain disorder (PEPD) [104,110]. Because of their monogenic nature, these conditions serve as valuable human models for understanding genetic pain mechanisms. At the cellular level, IEM mutations typically lower the threshold for NaV1.7 channel activation, boost ramp current amplitudes, and delay channel deactivation. Consequently, when expressed in DRG sensory neurons, these mutant variants drive hyperexcitability by decreasing current thresholds and accelerating firing rates [104,111]. Conversely, PEPD-associated mutations primarily disrupt the channel′s fast inactivation process, ensuring that a larger pool of functional channels remains available, increasing DRG neuron excitability [104,111]. Gain-of-function variants have also been linked to a more widespread condition: idiopathic small fiber neuropathy (SFN), which usually manifests in adulthood, with burning neuropathic pain as the primary symptom, often accompanied by signs of autonomic dysfunction [104,112].

At the opposite pole, loss-of-function mutations in NaV1.7 cause complete congenital insensitivity to pain, a very rare condition, unlike the congenital absence of hearing or vision. Individuals with biallelic null variants experience no pain whatsoever, tolerating bone fractures, dental procedures, burns, and even childbirth without discomfort. Aside from anosmia, these patients generally retain normal motor, cognitive, autonomic, and gastrointestinal function, reinforcing NaV1.7′s role as a peripheral pain channel and suggesting that its pharmacological inhibition could produce safe analgesia, without central side effects [60,104,113,114].

This created an effervescent interest for selective NaV1.7 inhibitors, and a number of them were developed and entered clinical development: Vixotrigine (BIIB074) [115], funapide (TV-45070) [116], GDC-0310 [117], PF-05089771 [118,119], AMG8379 [120], ralfinamide, benzazepinone, and others [116]. However, most often after initial high hopes, they disappointed in clinical trials, as discussed in [116]. The underlying drivers of these clinical failures are multifaceted, encompassing variables in target biology, medicinal chemistry, pharmacokinetics, and translational study design. Rather than attributing the disappointing outcomes to a single cause, it seems likely that a complex interplay among these multiple factors is responsible:

Probably the first and foremost is related to the (in)correct understanding of the target and its validity. Sodium channel blockers only interrupt nerve conduction, whereas available evidence indicates that the congenital painlessness of NaV1.7-null subjects seem to be related to a dual effect that includes enhanced opioid activity. NaV1.7 loss upregulates enkephalin production in sensory neurons (unlike NaV1.8 loss), and opioid blockade with naloxone abolishes the analgesic effect in both animal models and human subjects. In murine studies, blocking or knocking out both µ- and δ-opioid receptors eliminate the opioid-induced analgesia observed in NaV1.7-null mice (whereas κ-opioid receptor antagonists have no such impact) [121]. Mice lacking NaV1.7 preserve their nociceptor function; however, transmission at the central synapses of nociceptor terminals within the spinal cord is markedly attenuated through a mechanism dependent on opioid signaling (a reduction in pronociceptive serotonergic signaling through the 5-HT_4_ receptors has also been reported). This is why the resulting analgesia is largely reversed by central, but not peripheral, administration of opioid receptor antagonists [122,123]. These data indicate that selective NaV1.7 inhibitors cannot on their own reproduce the null phenotype observed in pain-insensitive human conditions; they might be potentiated, though, by co-administering opioids [124]. Nevertheless, this is not as attractive therapeutically, although if substantially lower doses are needed, it could still be of a high value; early evidence indicates that this does seem to be the case [125,126]). A related explanation, supported by limited evidence, is that lifelong NaV1.7 deficiency may trigger compensatory molecular and neuronal adaptations that cannot be reproduced by short-term pharmacological blockade. Such adaptations include the enhanced endogenous opioid signaling, as well as additional adaptive mechanisms, such as transcriptional or synaptic remodeling, which together contribute to their marked insensitivity to pain [127]. Deng et al. (2023) showed that enkephalin overexpression occurs selectively in cLTMR neurons, not universally across all neurons, and that this specific overexpression does not contribute to the analgesia observed after NaV1.7 genetic deletion [128]. In this context, Shields et al. (2018) claimed to have achieved analgesic effects within 1 h of dosing with blockade of NaV1.7 channels, indicating that pain thresholds can be elevated without requiring opioid upregulation [129].

Pain transmission relies on multiple sodium channel isoforms (Nav1.3, NaV1.7, NaV1.8, and Nav1.9), as well as other ion channels including calcium, potassium, and TRP channels. Therefore, selective NaV1.7 inhibition may prove inadequate for pain suppression if alternative channels compensate that inhibition. NaV1.8 and Nav1.9, for example, are co-expressed with NaV1.7 in peripheral nociceptors [130] and could partly compensate for NaV1.7 inhibition in certain pain states. This reveals a key challenge: blocking just NaV1.7 may be insufficient given the redundancy of sodium channels in nociceptors, yet blocking multiple channels simultaneously narrows the safety margin, as in the case of non-selective blockers.

The significant similarity in amino acid sequences across the various Nav subtypes poses a major challenge in identifying ligands that can selectively target just one subtype [53]. Early clinical drug candidates exhibited limited selectivity over off-target Nav isoforms, which is a critical safety liability. In the literature it has been speculated that selectivity ratios greater than 100-fold may be necessary to achieve efficacy without toxicity. Moreover, if subtler adverse effects like paresthesia or CNS disturbances appear before motor deficits, the selectivity threshold required for an adequate human safety margin could be substantially higher still [131]. Thus, designing analgesics that target sodium channels creates a dual challenge: compounds must possess a wide enough inhibitory profile to compensate for biological redundancy, while retaining the target selectivity required to prevent dose-limiting toxicity.

Only a subset of animal pain models actually rely on NaV1.7-mediated excitability. Robust NaV1.7 involvement is seen in thermal hypersensitivity (CFA) and capsaicin-induced mechanical allodynia. Instead, mechanical withdrawal tests (von Frey), formalin-evoked behaviors, and the majority of mechanical allodynia models, including neuropathic, inflammatory, and postoperative models, as well as electrical stimulation, show little or no dependence on NaV1.7 deletion [129].

Numerous early NaV1.7 drug candidates were designed to remain in the peripheral territories of the body, to prevent brain penetration and associated adverse effects. However, chronic pain is often driven by central sensitization, a hyperexcitable state of neurons in the spinal cord dorsal horn and brain. Because these selective inhibitors could not cross the BBB, they remained ineffective against the central drivers of chronic pain, leaving the underlying pathology virtually unaddressed. Experimental murine data have shown that spinal cord injury induces NaV1.7 upregulation in both superficial dorsal horn and DRG neurons. In these experiments, inhibiting NaV1.7 in both peripheral and spinal neurons was more effective at relieving mechanical pain than peripheral inhibition alone. This suggests that a BBB-permeable NaV1.7 blocker could offer therapeutic benefit for neuropathic pain in spinal cord injury patients [58,75,101]. Inhibiting NaV1.7 with compounds that cross the ΒBΒ is expected to cause anosmia [58]. In Nav1.7-null individuals, this anosmia results from a defect in synaptic transmission, not from a problem with odor detection. Olfactory sensory neurons respond normally to odors but cannot depolarize sufficiently to activate neurons in the olfactory bulb. This phenomenon is similar to the transmission defect that causes the analgesia associated with the Nav1.7 gene deletion [132]. While the analgesia associated with the Nav1.7 deletion depends on (or at least is influenced by) endogenous opioid signaling within nociceptive pathways, the anosmic phenotype involves a mechanism independent of opioids [122]. Inhibiting NaV1.7 with compounds that cross the ΒΒΒ may also lead to weight gain through blockade of NaV1.7 channels in hypothalamic neurons, although individuals with congenital NaV1.7 pain insensitivity do not exhibit this weight gain issue [58].

While rodent models offer crucial mechanistic understanding of NaV channels, their translational usefulness is limited. This is due to (1) species-specific channel biology, (2) dependence on stimulus-induced reflexive outcomes, (3) variations between acute and chronic pain mechanisms, and (4) pharmacokinetic differences that hinder direct application to human nociceptors and clinical pain conditions. The preclinical models used to support the clinical development of NaV1.7 inhibitors were rather inadequate to reflect the complexity of human pain disorders and these inadequacies have been analyzed in detail by others [133]. Beyond inherent interspecies differences, most studies employed young, genetically homogeneous male rodents, which poorly represent the heterogeneous patient populations enrolled in clinical trials. The available electrophysiological data indicate that resting-state voltage-dependent sodium currents in DRG neurons from mice not exposed to stimuli do not show substantial differences between the sexes in terms of activation properties or peak current density [134]. This suggests that the intrinsic biophysical properties of the channels are, to a large extent, comparable between the sexes. However, nociceptive signaling pathways upstream and downstream of NaV channels may differ significantly by sex: microglia-dependent sensitization predominates in males, while T-cell-mediated mechanisms are more prominent in females [135]. This suggests that system-level responses to sodium channel inhibition may differ even when channel-level effects are similar. From a clinical perspective, clinical trials of analgesics, including those involving nonselective sodium channel blockers and selective subtype inhibitors currently in early-stage development, rarely stratify efficacy or pharmacokinetics by sex. This is despite evidence that women often have higher plasma concentrations or altered volumes of distribution for lipophilic sodium channel blockers [136]. Taken together, these findings suggest that, although NaV1.7/NaV1.8 inhibition may function similarly at the single-cell level, sex-dependent differences could influence therapeutic response and deserve greater attention in future mechanistic and clinical studies.

Inflammatory pain models predominated in preclinical research, whereas clinical studies primarily targeted neuropathic pain, despite some evidence suggesting that NaV1.7 is less critical in this condition. Non-clinical programs often rely on evoked pain endpoints in animal studies, unlike clinical trials that assess average pain intensity. Additionally, they widely use single-dose administration, whereas clinical subjects receive repeated doses. Such factors limit the ability to detect tolerance or other time-dependent pharmacodynamic effects in non-clinical studies [133].

The first disappointing clinical trials involved the nonselective, state-dependent inhibitors with high plasma protein binding, and therefore they might have needed greater systemic exposure for efficacy. Evaluating plasma protein binding is difficult for lipophilic drugs, particularly when they are 99% or more bound. Additionally, unbound plasma drug concentrations may not accurately reflect true exposure levels within neuronal tissue [131]. For instance, two analogs designed for better membrane permeability achieved much higher DRG levels than others, but only one of these compounds was effective in the formalin model [137].

To mimic the gene knock-out effects seen in human pain insensitivity conditions, available evidence suggests that at least 80–90% NaV1.7 occupancy is needed. Achieving this is challenging if one considers additional challenges, such as tissue barriers (including the nerve sheath and BBB), fast unbinding kinetics, state-dependent channel behavior, and diminished drug potency when accessory subunits are present. Rodent model data suggest that plasma concentrations higher than 20–100x IC_50_ are necessary [131]. Enhanced therapeutic efficacy seems also associated with extremely slow dissociation kinetics from the target channel, as shown by mice data. Chronic administration was reported to increase compound potency about 10 times and produced sustained efficacy outlasting plasma clearance of the drug [138].

Some of them (such as PF-05089771) exhibit preferential binding to the channel′s *inactivated state* rather than to its resting or open conformations. Because the relative distribution of these conformational states in vivo remains poorly defined and is likely to differ across tissues and pathological pain conditions, predicting therapeutic efficacy is challenging. Consequently, one possible explanation for variable efficacy is that state-dependent inhibition complicates the assessment of target engagement under physiological and disease-relevant conditions [131].

In NaV1.8-deficient mice, increased NaV1.7 mRNA expression has been observed, suggesting that NaV1.7 may partially compensate for the loss of NaV1.8 [139]. However, it is entirely possible that other compensatory mechanisms, involving additional sodium channel subtypes or alternative signaling pathways, could also offset NaV1.7 inhibition. This could also explain the hypothesis formulated relatively long ago that there is an inverse relationship between NaV1.7 selectivity and analgesic potency. Highly selective inhibitors, such as protoxin II, produce weaker pain relief, whereas less selective antagonists (like CNV-1014802 and lidocaine), which target a broader range of sodium channels, show higher efficacy [59].

Chronic pain is not a homogenous entity, but rather a conglomerate of disorders driven by distinct underlying mechanisms, and this suggests that no single sodium channel target is likely to provide universal efficacy in chronic pain [58,101]. Including diverse pain phenotypes in clinical trials may have diluted efficacy signals, obscuring benefits confined to NaV1.7-driven subgroups. Therefore, to succeed, future drug development must adopt a precision medicine approach, mapping out the exact patient populations and pain etiologies where NaV1.7 inhibition is most effective [101,140]. Patient selection based on genetics, such as mutations in NaV1.7 channels (channelopathies) could be considered in certain trials [104]. But inconsistent efficacy of NaV1.7 inhibitors may partly reflect the dynamic regulation of NaV1.7 expression and function (e.g., trafficking, phosphorylation, membrane localization, gating). While congenital SCN9A mutations firmly establish the channel as a pain target, its activity in common chronic pain conditions is modulated by inflammatory mediators, nerve injury, and possibly a variety of epigenetic mechanisms. As a result, NaV1.7-mediated signaling may vary considerably among patients sharing the same clinical diagnosis. If this hypothesis is correct, future research may therefore benefit from identifying biomarkers of functional NaV1.7 activity rather than relying exclusively on disease categorization.

To understand the discrepancy between the effects of NaV1.7 inhibitors and genetic predictions regarding clinical efficacy, it is essential to distinguish between four distinct experimental contexts:

(a) **Congenital loss of function at the germline level.** The complete lifelong absence of NaV1.7 causes congenital insensitivity to pain (CIP). This phenotype is influenced by developmental plasticity, including upregulation of endogenous opioids (although the relative contribution of each mechanism remains unclear). Analgesia in mice and humans with a NaV1.7 gene deletion is substantially reversed by naloxone, suggesting an opioid-dependent suppression of neurotransmitter release in the dorsal horn, a situation distinct from that of deletions induced in adulthood. Thus, the analgesic effect in CIP reflects a systemic adaptation rather than an isolated loss of NaV1.7-mediated sodium conductance.
(b) **Adult-onset deletion.** Genetic deletion initiated in mature animals (e.g., [129]) bypasses development-related compensatory mechanisms, such as those involving the opioid system. However, it still removes the entire protein structure, affecting its non-conductive roles like scaffolding, protein-protein interactions, and local signaling complexes, which small molecules do not impact. Overexpression of pro-enkephalin (Penk) occurs only in cLTMR neurons (involved in low-threshold tactile sensation, not pain), and the analgesia following NaV1.7 ablation in adults is opioid-independent [128]. This contrasts with embryonic models in which opioid signaling contributes substantially to analgesia.
(c) **Acute pharmacological inhibition.** Small-molecule blockers selectively target the channel opening mechanism or conductance in a reversible and cell-state-dependent manner. As in situation (b) above, acute interaction with the target in adult tissues does not trigger the compensatory changes observed in embryonic models [128] (it does not rapidly increase the expression of endogenous opioids). However, in this case, the protein’s non-conductive roles are no longer disrupted, only its conductive ones. Any analgesic effect must result exclusively from a reduction in the initiation or propagation of the action potential in nociceptors.
(d) **Chronic pharmacological inhibition.** Prolonged exposure may introduce additional kinetic or traffic effects (slow dissociation, occupancy accumulation, possible changes in channel surface expression), but it does not alter the protein structure and is unlikely to reproduce opioid adaptations or those associated with the development of a germline null mutation. Chronic administration may, therefore, increase efficacy compared to acute administration, but its underlying mechanism remains substantially different from that of a genetic deletion of the channel.

Recognizing these differences may explain why one cannot expect acute or chronic small-molecule blockade in adults to automatically reproduce the complex, multisystemic analgesia of germline CIP.

## 5. Why NaV1.8 Changed the Success Equation

NaV1.7 is expressed at high levels not only in nociceptive dorsal root ganglion and trigeminal neurons but also in sympathetic ganglion neurons [130,141], reflecting its broader role across the peripheral autonomic and sensory systems. It was originally cloned from human neuroendocrine cells [142], its transcripts are predominant in rodent olfactory sensory neurons [143], and this is consistent with the anosmia observed in patients with loss-of-function SCN9A mutations [144,145]. NaV1.7 expression in sympathetic rather than sensory neurons has shown its important role in the development of chronic neuropathic pain after nerve injury [105], indicating that its analgesic relevance cannot be attributed to nociceptors alone. NaV1.8, by contrast, has a relatively narrower distribution, with expression largely restricted to peripheral sensory neurons, the majority of which respond to noxious stimuli. When NaV1.8 is deleted, spinal dorsal horn neurons show marked reductions in evoked activity in response to a range of mechanical stimuli, such as light touch (brush), moderate pressure (von Frey), and strong noxious input (pinch). This aligns with the known expression pattern of NaV1.8, which is found predominantly in small-diameter nociceptive afferents, not in the large-diameter low-threshold mechanoreceptors responsible for innocuous touch. Instead, thermal responses are unaffected (NaV1.8 is not required for heat-evoked nociception) [146].

While NaV1.8 is known to be predominantly expressed in small, unmyelinated sensory neurons that detect painful stimuli, Shields et al. (2012) [147] showed that 75% of DRG neurons express NaV1.8-Cre. This includes over 90% of nociceptive marker-positive neurons and, surprisingly, roughly 40% of myelinated A-fiber neurons. However, NaV1.8-Cre indicates a developmental lineage rather than real-time channel expression. Some NaV1.8-Cre-positive neurons in the adult may no longer express functional NaV1.8 protein, a caveat that complicates the interpretation of phenotypes derived from NaV1.8-Cre conditional knockout models [147]. Thus, while NaV1.8 offers a significantly narrower expression than NaV1.7 in the peripheral regions of the body (avoiding olfactory and neuroendocrine off-target sites), neither channel is limited to nociceptors.

Despite their frequent co-expression in nociceptive DRG neurons, NaV1.7 and NaV1.8 serve distinct but complementary functions in action potential generation and conduction. NaV1.7 operates principally as a threshold channel, becoming active at relatively hyperpolarized membrane potentials (half-maximal activation near −30 mV) and amplifying minor subthreshold depolarizations to promote action potential initiation. In contrast, NaV1.8 is active at more depolarized voltages (half-maximally activated at 0 mV) and displays relatively depolarized steady-state inactivation coupled with rapid recovery from inactivation. These properties permit a significant portion of channels to stay available even during prolonged depolarization [58,148,149]. Because of NaV1.8′s depolarized voltage-dependence, NaV1.8 channels are likely activated after NaV1.7 currents in response to stimuli. Thus, in contrast to NaV1.7, NaV1.8 is less likely to contribute to setting the threshold for generation of action potentials [148]. Therefore, NaV1.8 contributes substantially to the inward sodium current during the action potential upstroke in nociceptors and is critical for sustaining repetitive firing, especially under pathological conditions linked to chronic pain.

While NaV1.7 primarily controls the excitation threshold of nociceptors, NaV1.8 mainly dictates their capacity for sustained repetitive discharge, which is a key feature of persistent nociceptive signaling. Thus, its inhibition provides more direct control over pain signaling. In other words, because persistent pain is maintained by ongoing, repetitive ectopic discharge, inhibiting NaV1.8 may suppress the continuous firing itself rather than merely increasing the threshold required to initiate it [58,148,149]. NaV1.8 blockade causes a far greater suppression of repetitive firing than NaV1.7 inhibition. Although inhibiting NaV1.7 alters human DRG excitability, raising threshold and prolonging the refractory period, it has minimal impact on the ability of neurons to fire repetitively under strong stimulation [150]. This provides a plausible mechanistic advantage over NaV1.7 inhibition. The distinct functions of the two channels provide a reasonable mechanistic explanation for the clinical efficacy of selective NaV1.8 blockade with suzetrigine. Although both NaV1.7 and NaV1.8 contribute to varying degrees in different nociceptor populations and pain states, they should be regarded as complementary rather than as exclusive [58].

Moreover, while the opioid system and reduced serotonergic signaling appear to play to some extent a role in pain insensitivity adaptations related to NaV1.7 [123], there is currently no evidence that pain relief from NaV1.8 deficiency is due to increased endogenous opioid activity. Therefore, pharmacologically inhibiting NaV1.8 may more faithfully replicate the natural effects of reduced channel function.

The functional redundancy in the case of NaV1.8 blocking within nociceptors seems to be substantially limited, unlike NaV1.7 redundancy [151,152]. For instance, deletion of NaV1.8 eliminates TTX-resistant compound action potentials in distal C-fibers, indicating that no other sodium channel isoform compensates for its function at this site [151]. This diminished redundancy in peripheral pain signaling could theoretically increase the efficacy of targeting NaV1.8, unlike targeting NaV1.7. It could also explain suzetrigine’s success (we define ‘success’ in Section 6 as suzetrigine’s regulatory and mechanistic achievements, rather than its clinical superiority to opioids).

Nevertheless, these proposed explanations remain partly speculative, reflecting the current gaps in our understanding of how NaV1.7 and NaV1.8 differ functionally. Multiple lines of evidence suggest the relationship between these channels goes beyond a simple “threshold channel” (NaV1.7) and “firing channel” (NaV1.8) dichotomy. For instance, biallelic SCN9A (NaV1.7) loss-of-function mutations lead to congenital pain insensitivity. Instead, the rare biallelic SCN10A (NaV1.8) loss-of-function mutations documented to date have been tied to epilepsy-related conditions, including Lennox–Gastaut syndrome, infantile spasms, and autism spectrum disorder, rather than pain insensitivity [153,154]. At first glance at least, this seems inconsistent with NaV1.8 being the more critical player in pain. However, the very limited number of such patients, the predominance of neurological manifestations, and the potential for developmental or compensatory changes, preclude firm conclusions about the effects of complete NaV1.8 loss in humans. A SNP (rs6795970) at the level of *SCN10A* was reported to associate with higher thresholds for mechanical pain [155].

The development of many NaV1.8 inhibitors was halted after genetic studies associated SCN10A, the gene encoding NaV1.8, with cardiovascular disorders such as Brugada syndrome and sudden cardiac death. These findings raised concerns about the potential cardiac safety of this therapeutic strategy [127,156]. These concerns have been mitigated by the discovery of a hidden intronic promoter that generates a truncated C-terminal fragment known as SCN10A-short. Retaining only the last eight transmembrane segments of NaV1.8, this inactive variant acts as a functional modifier that chaperones or boosts the principal cardiac channel, Nav1.5. When this fragment is lost, the resulting drop in Nav1.5 current can trigger Brugada syndrome and sudden cardiac death. This dual neuro-cardiac role provides a coherent explanation for the mystery of why living individuals with bi-allelic NaV1.8 mutations and complete pain insensitivity cannot be found in human populations. Disrupting the gene does not just silence nociceptors, it also destabilizes heart development and can result in death [127,157].

Preclinical data also reveal substantial complexity. For instance, while one study suggested thermal responses were intact [146], an older study showed that homozygous NaV1.8 knockout mice exhibited (small) deficits in noxious heat responses and delayed development of inflammatory hyperalgesia [158], supporting a significant but non-exclusive role for NaV1.8 in nociception. Cold sensitivity is altered when either NaV1.8 or Nav1.9 is deleted alone, yet the combined loss of both channels leaves cold thresholds unchanged [159], pointing to functional redundancy and compensatory mechanisms within the sodium channel family. Collectively, these observations indicate that the relative contributions of NaV1.7 and NaV1.8 to nociception are context-dependent and incompletely understood, underscoring the need for caution when interpreting suzetrigine’s clinical success through a reductionist mechanistic framework.

A synthetic presentation of the mechanistic and translational differences between NaV1.7 and NaV1.8 channels is shown in Table 2.

## 6. Why Did Suzetrigine Succeed Where Previous NaV1.8 Inhibitors (and Most NaV1.7 Inhibitors) Did Not?

The word “success” employed in this paper might puzzle someone familiar with the fact that pain therapy is far from a solved problem. It is necessary to distinguish among three senses of “success” that are frequently conflated in the scientific discourse on Nav-channel analgesics. The first is *mechanistic success*: demonstration that a drug engages its intended molecular target with sufficient potency and selectivity to produce a measurable physiological effect (case in point: reduced excitability of human peripheral nociceptors via state-dependent NaV1.8 inhibition). A second sense refers to *regulatory and clinical-trial success*: achievement of statistically significant, clinically meaningful outcomes against pre-specified endpoints sufficient to satisfy regulatory authorities and, consequently, getting regulatory approval. A third and probably most demanding sense of the word is *therapeutic success* in the broader public-health sense: displacement (or at least substantial reduction) of opioid analgesics in clinical practice. This requires efficacy and tolerability sufficient to shift prescribing practice rather than merely supplement current therapies.

Suzetrigine has clearly achieved the first two of these three benchmarks: it validated peripheral NaV1.8 blockade as a druggable, selective mechanism in humans, and it cleared a regulatory pathway to approval. As to the third sense (therapeutic success at the level of opioid replacement) it may be premature to affirm it or deny it, but currently available data suggest that at best it is only partially achieved. Understanding the different senses of “success” is essential to avoid overstating the therapeutic role of suzetrigine and to calibrate expectations. When we talk about “success” in this paper, we rather have in mind the first two senses. We believe that suzetrigine may be just the first within a class (or several classes) of new analgesics, which should be superior to it. The suzetrigine regulatory approval does not mean that NaV1.8 was simply the “right” target whereas NaV1.7 was not. Rather, it illustrates the idea that successful (analgesic) drug development is the result of an interplay between target biology, medicinal chemistry, pharmacokinetics, clinical development, and a savvy regulatory strategy.

Selective NaV1.8 inhibition had been pursued for more than a decade before suzetrigine reached the market. Several selective inhibitors (e.g., A-803467 [164], PF-01247324 [165], and VX-150 [166]) exhibited encouraging preclinical efficacy, thereby validating NaV1.8 as a pharmacologically relevant target (Table 3). However, poor oral bioavailability and high protein binding prevented A-803467 [162] from advancing beyond preclinical testing. PF-01247324 achieved high oral bioavailability (F = 91%) and demonstrated efficacy in both rodent pain models and ex vivo human DRG neuron electrophysiology; yet it likewise did not progress to clinical development [162], although the precise reason for this remains unknown. Only VX-150 advanced to Phase 2 trials, where it showed analgesic activity but was ultimately discontinued. This discontinuation seems to be related more to its low potency rather than inherent lack of efficacy (high doses were necessary to get a moderate analgesic effect) [167,168]. The approval of suzetrigine seems more a peak of progressive advances in NaV1.8 drug discovery and clinical translation than as a simple consequence of target selection alone. Its success deserves broader analysis to understand what distinguish it from earlier members of the same pharmacological class.

Both suzetrigine and VX-150 (which was its precursor) work by state-dependent inhibition, meaning they mainly bind to the inactivated form of the channel instead of its resting or open states. This is important because peripheral nociceptors depolarize and inactivate their sodium channels more often during abnormal, high-frequency firing than during normal activity. State-dependent binding lets the drug target and act on hyperactive nociceptors while leaving normal neuronal function in the periphery unaffected [169].

This kinetic selectivity is enhanced by a high level of molecular selectivity. In vitro pharmacological studies showed that suzetrigine is over 31,000 times more selective for NaV1.8 compared to other sodium channel types and unrelated molecular targets [170]. For comparison, VX-150, considered itself a highly selective NaV1.8 blocker, was over 400 times more selective compared with other Nav isoforms [171].

With its very high specificity, at clinically relevant concentrations suzetrigine causes less than 0.1% inhibition across all other VGSC isoforms. Furthermore, safety screens against 180 non-Nav human targets, including 44 distinct receptors and channels traditionally implicated in substance abuse liability, demonstrated a greater than 600-fold selectivity margin relative to estimated therapeutic exposures. This clean off-target profile (with no impact on Nav1.5) supports an absence of cardiovascular adverse effects; non-clinical safety evaluations revealed no adverse impact on blood pressure or electrocardiogram (ECG) parameters. Repeat-dose toxicology studies in non-human primates across a 9-month period at or above recommended human exposure thresholds produced no quantitative or qualitative abnormalities on surface ECGs [170].

The mechanistic and regulatory success of suzetrigine has also been underpinned by its favorable pharmacokinetic profile, which successfully overcome several PK barriers known for other sodium channel blockers. While earlier non-selective alternatives like intravenous lidocaine required continuous systemic infusion, suzetrigine achieves robust oral bioavailability, with fast absorption, allowing convenient oral dosing (but it is still protein-bound in a proportion of 99%) [172]. Moreover, the molecule is chemically engineered to have its distribution restricted to the peripheral region. Its poor blood-brain barrier permeability ensures it cannot easily penetrate the central nervous system, and thus minimizes the risk of CNS side effects [173]. The high plasma protein binding gives suzetrigine a long half-life (~23.6 h) and steady systemic levels, supporting stable concentrations across the dosing window and making twice-daily dosing practical [173].

The choice of the trial design and the target population also probably played a role in suzetrigine’s success. Its pivotal trial enrolled patients undergoing bunionectomy or abdominoplasty. These are two procedures widely used in analgesic development because they generate a standardized, predictable, and time-limited nociceptive pain signal for both a soft-tissue (abdominoplasty) and a hard-tissue/bone (bunionectomy). This is a sharp contrast to the heterogeneous chronic and neuropathic pain populations (diabetic neuropathy, central post-stroke pain, mixed-etiology neuropathic pain) that had previously undermined both the NaV1.7 selective-inhibitors and lamotrigine. In these cases, variable disease mechanisms diluted efficacy signals making reaching the primary endpoint harder to achieve [13,83,116,174]. Both Phase 3 trials (NAVIGATE 1, for bunionectomy and NAVIGATE 2 for abdominoplasty) were placebo- and active-controlled, randomizing patients 2:2:1 to suzetrigine, hydrocodone bitartrate/acetaminophen, or placebo. The primary endpoint (time-weighted sum of pain intensity difference over 48 h—SPID48) was met with statistical significance in both trials (against placebo). However, neither trial achieved its key secondary endpoint of superiority over hydrocodone/acetaminophen on SPID48, though suzetrigine did show a more rapid onset of clinically meaningful pain relief than placebo [175]. By evaluating suzetrigine directly against opioid control rather than placebo alone, the clinical program generated the head-to-head efficacy context needed to support its clinical positioning as a non-opioid alternative, even in the absence of superiority. A detailed quantitative analysis of suzetrigine’s treatment effect sizes and confidence intervals is beyond the scope of this mechanistic review; readers interested in comprehensive efficacy data should consult the Phase 3 trial publications and available meta-analyses.

VX-150 had already demonstrated statistically significant relief of acute pain versus placebo in its own phase 2 bunionectomy trial (which also included a hydrocodone/acetaminophen arm, as a reference rather than a formal comparator). However, VX-150 required a substantial oral dose (1500 mg loading, then 750 mg every 12 h), reflecting comparatively modest potency and selectivity for NaV1.8. Besides, its adverse events, including headaches, were reasons for stopping further development [176]. Rather than advancing this molecule directly to Phase 3, Vertex pursued suzetrigine, while preserving the same trial architecture, reflecting continuity in trial design rather than a redesign dictated by failure. Not only did the phase II success of VX-150 prepare the ground for suzetrigine clinical program, but it also confirms the target validity by obtaining success with a more selective molecule for the same target.

Finally, suzetrigine regulatory approval took place in a specific policy environment. In response to the escalating opioid crisis, the FDA has long supported the development of non-opioid analgesics for acute pain management. This is part of its Overdose Prevention Framework and reflects a wider institutional position influenced by ongoing opioid-related morbidity and mortality [177]. Within this environment, suzetrigine′s development benefited from Fast Track and Breakthrough Therapy designations from the FDA for treating moderate to severe acute pain [178]. The non-addictive pharmacological profile of suzetrigine shaped not only the regulatory pathway but suzetrigine′s entire clinical positioning. It has a decreased likelihood of drug dependence as compared to opioids and was not associated with respiratory depression or sedation [172], in contrast to the hydrocodone bitartrate/acetaminophen comparator arm in its pivotal trials. Its positioning relative to existing non-opioid analgesics (NSAIDs and acetaminophen) refers to a difference of mechanism rather than of magnitude. Comparative efficacy data suggest that suzetrigine exhibits lower clinical potency than hydrocodone-acetaminophen in ambulatory post-surgical settings. This is because a high-dose regimen of suzetrigine (a 100 mg loading dose followed by 50 mg twice daily) yielded analgesic effects equivalent only to a low-dose combination of hydrocodone and paracetamol (5/325 mg, four times daily) [179]. It thus offers a mechanistically distinct, non-addictive option for patients and prescribers interested in avoiding opioid-related risks, rather than a more effective option to existing non-opioid standards of care with respect to analgesic magnitude.

Thus, suzetrigine’s approval cannot be attributed exclusively to the NaV1.8 target selection. It rather is the cumulative result of several factors:

• Substantially better molecular with a clean off-target profile (including avoidance of other NaV channel inhibition);
• State- and use-dependent inhibition;
• Minimal CNS penetration (confinement to the peripheral compartment);
• Good oral bioavailability;
• A sufficiently long half-life (related to high plasma protein binding) allowing twice-daily administration;
• Deliberate choice of homogenous pain models for the clinical trials;
• A favorable regulatory environment.

Coming back to the three success meanings, by the first criterion suzetrigine is unquestionably successful, having become the first selective voltage-gated sodium channel inhibitor approved for the treatment of acute pain. By the second, it has demonstrated statistically significant and clinically relevant analgesic efficacy with a favourable safety profile (no respiratory depression, abuse potential, or gastrointestinal adverse effects typically associated with opioids). However, the third criterion is not convincingly satisfied. Suzetrigine does not reproduce the profound analgesia observed in congenital pain insensitivity or the degree of pain relief achievable with potent opioid analgesics. This is consistent with the complementary physiological roles of NaV1.7 and NaV1.8. In the pivotal postoperative pain studies, suzetrigine produced significant reductions in pain intensity over placebo, yet the magnitude of analgesia was similar to that of hydrocodone/acetaminophen at low doses. Several voices have questioned the efficacy data supporting suzetrigine’s use in pain therapy up to date [180,181,182] and there is no doubt that better options will get approved in a nearer or farther future. Suzetrigine does not seem to be the end of the road, but rather the first step in a longer road, leading to good pain control without opioids.

## 7. Conclusions

Pharmacological research has attempted for over three decades to selectively target sodium channels for analgesic purposes. Although NaV1.7 channels seemed an ideal target, with excellent molecular and clinical validity, translation efforts have remained elusive. NaV1.8 channels and advancements in drug design created a first success story. For the first time in therapy, a non-opioid with a different mechanism than targeting cyclooxygenases has achieved regulatory approval for pain treatment. Based on what we know today, one is tempted to speculate that a second generation of NaV1.8 blockers will be developed in the future. These could have altered PK or PD properties and could be state selective or use dependent. Nav.1.7/1.8 dual blockers, a combination of NaV1.7 and NaV1.8 blockers, as well as other potential combinations (e.g., NSAIDs plus NaV1.8 blockers) might also emerge in the future, offering better options in controlling pain. Of course, other therapeutic targets continue to be explored beyond VGSCs.

## Figures and Tables

**Table 1:** Non-selective sodium channel inhibitors and their limitations in pain therapy.

Drug(s)	Mechanism of Action	Efficacy/Evidence	Limitations/Side Effects	References
Carbamazepine (and oxcarbazepine, eslicarbazepine)	Blocks voltage-gated sodium channels (prefer inactivated state); modulates GABA, interferes with glutamate (NMDA), regulates central sensitization. Higher affinity for inactivated channels (carbamazepine/oxcarbazepine > 10-fold; eslicarbazepine ~3-fold vs. carbamazepine). No Nav isoform selectivity.	Used as first line treatment in trigeminal neuralgia. Some efficacy in chronic neuropathic pain (limited quality trials); oxcarbazepine minimal/no benefit in diabetic neuropathy trials.	66% adverse reactions (vs. 27% placebo); short trials, poor reporting.	[77,78,79,80]
Phenytoin (systemic)	Sodium channel blocker	Indicated for trigeminal neuralgia (standalone/adjunctive, especially acute) and severe neuropathic pain needing rapid analgesia. No good-quality trials for chronic use.	Unfavorable side-effect profile limits clinical use.	[81,82]
Lamotrigine	Sodium channel blocker (non-selective, crosses BBB)	No convincing evidence of efficacy for acute/chronic pain (200–400 mg/day).	Common neurological side effects (dizziness, ataxia, diplopia, somnolence, nausea). Likely saturates CNS channels before peripheral effect.	[83,84]
Lacosamide	Promotes the slow inactivation phase of sodium channels.	Beneficial results for certain patients suffering from trigeminal neuralgia. Modest reduction in neuropathic pain. Preliminary, observational, limited evidence.	Sleepiness/somnolence (24.6%); dizziness (21.7%); instability (2.5%); first-degree atrioventricular block (1.7%); inattention (1.7%). High dropout rate in several clinical trials.	[85,86]
Valproate	Sodium channel blocker, inhibition of GABAergic and glutamatergic systems	May reduce pain in diabetic neuropathy, and divalproex sodium may reduce pain in post-herpetic neuralgia. Insufficient evidence.	Limited supportive data.	[71]
Topiramate	Blocking voltage-dependent sodium channels, boosting the effects of GABA at certain GABA receptors, and blocking non-NMDA glutamate receptors	No evidence of benefit in diabetic neuropathic pain.	High withdrawal rate due to adverse events (27% vs. 8% placebo at 400 mg/day).	[72,87]
IV Lidocaine (and oral mexiletine)	Sodium channel blockade	Used off-label since 1980s; some efficacy in refractory/chronic neuropathic pain (incl. diabetic); short-lived effects. Similar safety/efficacy to carbamazepine, gabapentin, etc. Mexiletine superior to placebo in meta-analysis. Pretreatment with lidocaine infusions is useful in predicting which patients are likely to adhere to mexiletine therapy	Inconsistent evidence; short duration; requires IV access & monitoring; cardiac/neurologic toxicity risk. Mexiletine: modest VAS reduction (~11 points), high dropout (50% by 43 days; <20% at 1 year).	[88,89,90,91,92,93,94,95]
Flecainide	Sodium channel blocker	Not used for pain therapy, due to its safety profile.	Narrow therapeutic index (0.2–1.0 mcg/mL); risk of severe cardiac toxicity (wide-complex tachycardia, VF, AV block, bradyarrhythmia, asystole).	[96]

**Table 2:** Mechanistic and Translational Differences Between NaV1.7 and NaV1.8 Voltage-Gated Sodium Channel Isoforms.

Comparison Parameter	Nav 1.7 (SCN9A)	NaV1.8 (SCN10A)	Clinical/Translational Implication
Anatomical and tissue expression	Widely expressed across peripheral tissues, especially abundant in nociceptive DRG and sympathetic ganglion neurons [160], but also detected in neuroendocrine and olfactory sensory cell populations [111].	Peripherally restricted to small-to-medium nociceptive DRG and trigeminal neurons [161]; full-length functional NaV1.8 protein is absent from CNS, heart, and skeletal muscle; SCN10A-derived truncated transcripts (e.g., SCN10A-short) may be present but they do not form NaV1.8 channels [157,162].	Targeting NaV1.8 reduces the likelihood of off-target effects, including NaV1.7-associated anosmia, owing to its more restricted tissue distribution.
Primary biophysical and electrophysiological role	Threshold Channel: Activates at relatively hyperpolarized potentials; amplifies weak, subthreshold generator potentials to drive the neuron toward its firing threshold [72,73,74].	Driver Channel: Activates at more depolarized voltages; exhibits depolarized steady-state inactivation and rapid recovery from inactivation. Drives the majority of the inward current during the action potential upstroke [72,73,74].	While NaV1.7 is responsible for triggering the initial action potential, NaV1.8 is essential for maintaining prolonged neuronal excitability. Blocking NaV1.8 substantially reduces the rapid, repetitive nerve firing required to sustain both acute and chronic pain states *.
Opioid system interdependence	Dependent: The profound analgesia observed in genetic loss-of-function (CIP) mutations seems driven by elevated enkephalin expression and increased opioid signaling in the CNS, an effect that can be blocked by naloxone administration [121].	Largely independent: Pain relief or reduced excitability stemming from channel deficiency or blockade does not rely on the opioid signaling systems [163].	Simply pharmacologically blocking NaV1.7 alone does not reproduce the same effects as genetic deletion without opioid co-activation. NaV1.8 inhibitors, instead, do closely mimic the natural low-function phenotype.
Functional redundancy and target compensation	High(er) Redundancy: Peripheral nociceptors express additional sodium and calcium channels that can readily compensate and maintain action potential conduction when NaV1.7 is selectively inhibited [130].	Low(er) Redundancy: Removing or pharmacologically blocking the channel abolishes tetrodotoxin-resistant (TTX-R) compound action potentials in distal C-fibers, indicating that other isoforms cannot make up for its absence [151].	High(er) redundancy undermines NaV1.7 as a monotherapy target. Low(er) redundancy makes NaV1.8 a far more dependable single-point bottleneck for pain signals.
Clinical efficacy and regulatory outcomes	Repeated Clinical Failures: Several selective small-molecule inhibitors have failed to demonstrate efficacy in clinical trials [116].	Regulatory Breakthrough: Suzetrigine, a selective inhibitor, reached a significant milestone in clinical success by providing clinically relevant and statistically significant relief from acute pain during Phase 3 trials, paving the way for regulatory approval.	Focus moved from adjusting the excitability threshold via NaV1.7 to directly suppressing the persistent, abnormal pacemaker-like firing driven by NaV1.8.

* These descriptions are general functional tendencies rather than strict mechanistic separations, given that NaV1.7 and NaV1.8 engage dynamically during overlapping phases of nociceptor excitability.

**Table 3:** Comparative overview of major NaV1.8 inhibitors, highlighting key pharmacological properties, preclinical performance, and clinical development outcome.

Compound	Selectivity	State-Dependence	BBB Penetration	Preclinical Analgesia	Clinical Stage	Development Outcome
A-803467	Over 100-fold selectivity against human Nav1.2, Nav1.3, Nav1.5, and Nav1.7	Reverse use-dependence (unblocking during depolarization, cumulatively enhanced by repeated depolarizations), state-dependence	Limited (based on limited evidence)	Robust rodent efficacy in several models, but not all (not active in formalin-induced or acute thermal pain).	Never advanced	Discontinued for poor oral bioavailability and high protein binding
PF-01247324	Over 50-fold selectivity for Nav1.8 against Nav1.1, Nav1.5, and Nav1.7	State-dependent, use-dependent	Good (non-clinical evidence)	Efficacy in both rodent pain models and ex vivo human DRG neuron electrophysiology	Never advanced	Discontinued (precise reason unknown)
VX-150	Over 400-fold potency against other Nav subtypes	Reverse-use dependence, state-dependence	Limited, if any	Good preclinical efficacy (limited public evidence)	Phase 2	Program halted (likely due to low potency, rather than inefficacy)
Suzetrigine	Over 31,000 -fold selectivity against other Nav types and molecular targets	Reverse-use dependence, state-dependence	Negligible	Strong efficacy	Phase 4 (post-marketing)	Regulatory approval

## Data Availability

No new data were created or analyzed in this study. Data sharing is not applicable to this article.

## Abbreviations

The following abbreviations are used in this manuscript:

A-fiber	Myelinated sensory fiber
BBB	Blood–brain barrier
C-fiber	Unmyelinated nociceptive fiber
CFA	Complete Freund’s adjuvant
CIP	Congenital insensitivity to pain
cLTMR	C-low-threshold mechanoreceptor
CNS	Central nervous system
COX	Cyclooxygenase
DRG	Dorsal root ganglion
ECG	Electrocardiogram
FDA	U.S. Food and Drug Administration
F	Bioavailability
HEK293	Human embryonic kidney 293 cells
IEM	Inherited erythromelalgia
NaV	Voltage-gated sodium channel
NMDA	N-methyl-D-aspartate receptor
NSAID	Non-steroidal anti-inflammatory drug
PAR-2	Protease-activated receptor 2
PD	Pharmacodynamics
PEPD	Paroxysmal extreme pain disorder
PK	Pharmacokinetics
PN1	Alternative name for Nav1.7
SCN	Sodium channel gene family prefix
SFN	Small-fiber neuropathy
SPID48	Time-weighted sum of pain intensity difference over 48 h
TCAs	Tricyclic antidepressants
TRP	Transient receptor potential channels
TRPV1	Transient receptor potential vanilloid 1
TTX	Tetrodotoxin
TTX-R	Tetrodotoxin-resistant sodium channels
TTX-S	Tetrodotoxin-sensitive sodium channels
VAS	Visual analogue scale
VGSC	Voltage-gated sodium channel
WNT	Wnt signaling pathway
